# Association between pre-pregnancy body weight and dietary pattern with large-for-gestational-age infants in gestational diabetes

**DOI:** 10.1186/s13098-019-0463-5

**Published:** 2019-08-22

**Authors:** Ana Munda, Marjanca Starčič Erjavec, Katja Molan, Jerneja Ambrožič Avguštin, Darja Žgur-Bertok, Draženka Pongrac Barlovič

**Affiliations:** 1 Department of Psychology, University of Ljubljana, Faculty of Arts, Ljubljana, Slovenia; 2 Department of Biology, University of Ljubljana, Biotechnical Faculty, Ljubljana, Slovenia; 3 Department of Endocrinology, Diabetes and Metabolic Diseases, University Medical Centre, Ljubljana, Slovenia; 4 Faculty of Medicine, University of Ljubljana, Ljubljana, Slovenia

**Keywords:** Gestational diabetes, Obesity, Large-for-gestational-age (LGA), Personality

## Abstract

**Background:**

Both obesity and gestational diabetes (GDM) are associated with adverse outcomes. Diet during pregnancy impacts weight gain and fetal growth. Therefore, we aimed to explore non-pharmacological treatment success depending on pre-pregnancy body weight and its association with large for gestational age (LGA) infants in women with GDM.

**Methods:**

In our observational study we investigated 57 singleton pregnant women with GDM. All women received standard treatment, including healthy diet education and regular medical checkups. Data were collected through blood analysis, medical records and questionnaires assessing diet before conception and during pregnancy. Differences in dietary patterns were compared in normal weight and overweight/obese group using Mann–Whitney U, Wilcoxon Signed Rank Test or Kruskal–Wallis test, as appropriate. Logistic regression was used for prediction of LGA. p-value less than 0.05 was used for statistical significance.

**Results:**

Preconceptionally, the Mann–Whitney U test showed that the normal-weight group (n = 41) more frequently consumed fruits (*U *= 116.5, *p *< 0.001), eggs (*U *= 189.5, *p *= 0.02), cheese (*U *= 148.0, *p *= 0.003) compared to the overweight/obese group (n = 16), that consumed more beef (*U *= 407.0, *p *= 0.03) and low-calorie beverages (*U *= 397.0, *p *= 0.05). During pregnancy both groups improved their diet, with no differences detected. Personality types differed only preconceptionally with regard to healthy diet. Excessive gestational weight gain did not significantly differ between body-weight groups (16.6% vs. 23.1%), neither did the incidence of LGA infants (46.2% vs. 43.8%). Significant predictors of LGA were paternal height (OR = 1.12, 95% CI 1.01–1.23), 3rd trimester HbA1c (OR = 0.50, 95% CI 0.26–0.97), unemployment (OR = 4.80, 95% CI 1.12–20.61) and diet improvement during pregnancy (OR = 1.19, 95% CI 1.02–1.39). After adjustment improvement in diet was no longer a significant predictor for LGA.

**Conclusion:**

Even though dietary patterns of the participants significantly improved during pregnancy, LGA infants were born independently of pre-pregnancy weight or diet and despite good glycemic control. Further research is needed to explore social determinants of health and whether solutions outside the health sector could provide efficient means in preventing adverse pregnancy outcomes as well as improving metabolic health.

## Background

The epidemic of obesity and the growing incidence of diabetes are global public health issues. Obesity affects both sexes and all age groups. On a global scale, in 2016 40% of women and 39% of men were overweight, while 11% of men and 15% of women were obese. Excessive weight and obesity are also increasing among children and adolescents with and 18% prevalence of obesity among the 5 to 19 year age group [1]. Obesity is transmitted from generation to generation and represents an intergenerational vicious cycle [2]. As over 25% of women in their reproductive years prior to conception are overweight (17%) or obese (8%) [3], special attention should be paid to this phenomenon. Maternal obesity is associated with a higher risk of gestational diabetes mellitus (GDM) [4]. The incidence of GDM is growing worldwide [5] and in Europe the prevalence varies between 2 and 6% [6]. Meta-analysis showed that the risk of developing GDM is two times higher among overweight women and four times higher among obese women, compared to pregnant women with normal weight [7]. GDM and obesity are associated with a number of risks for complications during pregnancy, delivery and later in life for mother and offspring [8, 9]. The most common complication is birth of infants that are born large for gestational age (LGA), which prevalence is estimated at 18.3% [10]. LGA infants have a higher risk of obesity, type 2 diabetes and cardiovascular diseases later in life [11]. Decreasing the incidence of LGA could therefore limit the obesity epidemic.

GDM treatment is usually focused on nutrition and/or physical activity with many times inconsistent results [12–14]. This may be because often glycemic control is overemphasized compared to the effects of obesity or gestational weight gain on negative pregnancy outcomes in everyday clinical practice. Moreover, personality traits and behaviors, which may lead to obesity, are often neglected [15, 16]. Therefore, the main aim of our study was to assess whether the success of the treatment of gestational diabetes differs in women with obesity/overweight compared to normal weight women. Since the mainstay of the GDM treatment is dietary intervention, we sought to investigate whether changes in dietary pattern differs according to the BMI before pregnancy. In addition, we were interested whether personality types impact dietary change made by women with GDM. Since prevention of LGA infants is one of the main goals of the GDM treatment, our third aim was to explore whether change in diet pattern had an effect on the incidence of LGA infants.

## Methods and materials

### Study design and sample

Women treated for GDM in 2017 in an outpatient clinic at the University Medical Center Ljubljana were invited to participate in our longitudinal study. Fifty-seven consecutive women responded to our invitation and gave consent to participate. Diagnosis of GDM was based on a 3 h 75 g glucose tolerance test (OGTT).

The participants received standard treatment in our outpatient clinic for pregnant women with GDM, which includes education on GDM and healthy diet. They were encouraged to keep a diet diary, monitor blood glucose levels and were regularly clinically examined with body weight measurement at each encounter at our clinic. Data were collected on three occasions: in the 2nd trimester, 3rd trimester and after delivery via an e-mail. In addition, data on pre-pregnancy body weight and weight at the time of delivery were collected from gynecological files. Excessive gestational weight gain was calculated based on IOM guidelines [17]. Fasting venous blood was sampled for the measurement of serum glucose, HbA1c, cholesterol and triglycerides using standard validated laboratory techniques.

### Measures

Patients completed survey questionnaires regarding their lifestyle pre-conceptionally and during pregnancy. A short questionnaire that was developed by the Slovenian National Institute of Public Health was used as a measure of adherence to the principles of a healthy diet (Additional file 1: Table S1) [18]. Cronbach’s alpha was acceptable for measurements performed pre-gravidally and during pregnancy (α_1_ = 0.79, α_2_ = 0.70, respectfully). In addition, a frequency of food consumption list was used to assess how often participants consumed particular foods. Furthermore, we evaluated how often complex carbohydrates were chosen instead of simple ones. Single-item measure for assessing frequency of fried food consumption and number of daily consumed meals were also included. Additionally, demographic variables such as age, level of education, living environment, marital status, employment status (employed/unemployed), religious affiliation, mother’s height and weight prior to conception, father’s height and weight, as well as mother’s and father’s birth weight were collected.

Infant’s birth weight and gestational age were extracted from birth records. We defined large for gestational age (LGA) as a birth weight at or above 90^th^ percentile for specific gestational age, based on a fetal weight equation [19].

The employed personality test [20] is an adaptation of the Persona personality test, that encompasses 34 items in pairs of opposite words. This test was used because it can be easily performed during routine clinical visits and can be rapidly analyzed on the spot by the nurse educators. Personality types determined by this test are based on two dimensions; the level of emotion (expressive or reserved) and the degree of power (directive or compliant). According to these two dimensions, the test divides people into four personality types; promoter (directive and expressive, interpersonal need is impact), facilitator (compliant and expressive, interpersonal need is membership), controller (directive and reserved, interpersonal need is achievement) and analyzer (compliant and reserved, interpersonal need is security).

### Statistical analyses

Analyses were carried out using SPSS version 22. Most of the variables were not sufficient for the assumptions of parametric statistics, so we used non-parametric tests. Mann–Whitney U test was used to compare two independent groups (mainly normal weight and overweight/obese group) and for comparing more than two independent groups Kruskal–Wallis test was conducted. Wilcoxon Signed Rank was used to assess differences from pre-pregnancy to state during pregnancy within each group.. Univariate and multivariate linear logistic regression was used for predicting the LGA. We used a standard way of reporting mean ± SD. p-value of less than 0.05 was used as a limit of statistical significance.

## Results

### Characteristics of the sample

The final sample comprised 57 maternal-infant pairs. Table 1 presents characteristics of patients included in the study altogether and according to the BMI status. Participants age ranged from 22 to 42 (31.4 ± 5.1). There were 41 women with normal pre gravid body mass index (BMI), (age: 31.7 ± 5.5; BMI: 22.1 ± 0.2) and 16 overweight (*n *= 9) or obese participants (*n *= 7). Their BMI ranged from 25.3 to 39.0 (age: 31.89 ± 4.2; BMI: 31.2 ± 1.2). BMI-based groups did not differ significantly on demographics (Table 2).

**Table 1:** Characteristics of patients included in the study altogether and according to the BMI status

Demographic variables	Normal weight (*N* = 41) *M* ± *SD* *n* (%)	Overweight and obese (*N* = 16) *M *± *SD* *n* (%)	Total (*N* = 57) *M* ± *SD* *n* (%)
Education			
Secondary	12 (30.8)	5 (31.3)	17 (30.9)
Vocational/BA	11 (28.2)	6 (37.5)	17 (30.9)
MA/PhD	16 (41.0)	5 (31.3)	21 (38.2)
Living environment			
Urban	19 (48.7)	6 (37.5)	25 (45.5)
Suburban	8 (20.5)	6 (37.5)	14 (25.5)
Rural	12 (30.8)	4 (25.0)	16 (29.1)
Employment			
Employed	29 (74.4)	12 (75.0)	41 (74.5)
Unemployed	10 (25.6)	4 (25.0)	14 (25.5)
Parity			
0	21 (55.3)	5 (31.3)	26 (48.2)
1	14 (36.8)	7 (43.8)	21 (38.9)
2	3 (7.9)	4 (25.0)	7 (13.0)
Marital status			
Single	1 (2.6)	0 (0)	1 (1.8)
Married	19 (48.7)	10 (62.5)	29 (52.7)
Non-marital partnership	19 (48.7)	6 (37.5)	25 (45.5)
Pregravid smokers	6 (15.4)	4 (25.0)	10 (18.2)
Personality types			
Promoter	6 (19.4)	3 (21.4)	9 (20.0)
Facilitator	18 (58.1)	8 (57.1)	26 (57.8)
Controller	0 (0)	0 (0)	0 (0)
Analyzer	7 (22.6)	3 (21.4)	10 (22.2)
Hba1c_2ndtrimester_ (%)	4.96 ± 0.06	4.96 ± .10	4.96 ± 0.29
HbA1c_3rd trimester_ (%)	5.11 ± 0.39	5.23 ± 0.15	5.15 ± 0.34
Triglycerides (mmol/l)	1.77 ± 0.62	1.97 ± 0.55	1.83 ± 0.60

**Table 2:** Disparities among BMI groups and correlation between diet variables and BMI/GWG preconceptionally/during pregnancy

	Pre-conceptionally								Pregnancy							
	Normal weight		Overweight and obese		*U*	*p*	Total		Normal weight		Overweight and obese		*U*	*p*	Total	
	Median	*r*_*s*_ with BMI	Median	*r*_*s*_ with BMI			Median	*r*_*s*_ with BMI	Median	*r*_*s*_ with GWG	Median	*r*_*s*_ with GWG			Median	*r*_*s*_ with GWG
Healthy eating score	46.0	0.308	45.0	0.238	246.0	0.524	45.5	0.102	53.0	− 0.065	52.0	0.293	129.0	0.732	53.0	− 0.008
Number of meals per day	3.0	− 0.055	3.0	0.284	281.5	0.819	3.0	− 0.031	5.0	0.110	5.0	0.020	154.5	0.890	5.0	0.093
Complex carbohydrates	1.0	0.241	1.0	0.661*	217.5	0.939	1.0	0.186	3.0	0.197	3.0	− 0.432	81.5	0.823	3.0	0.084
Fried food	3.0	− 0.187	3.0	*−* 0.546*	366.0	0.227	3.0	*−* 0.268*	3.0	− 0.016	2.0	0.125	199.0	0.131	3.0	0.170

Italics text indicates statistically significant correlation

GWG: gestational weight gain; *r*_*s*_ with BMI: Spearman correlation with BMI; *r*_*s*_ with GWG: Spearman correlation with weight gain

*p < 0.05 level

### Differences in eating patterns in women with GDM

Before pregnancy the normal BMI group more frequently consumed fruits, eggs and cheese or cottage cheese than overweight and obese women (see Table 3) A Wilcoxon signed rank test indicated that all participants introduced positive changes in variables measuring nutrition, including an improvement in a healthy eating score (*Z *= 592.5, *p *< 0.001), consuming complex carbohydrates more often (*Z *= 194; *p *= 0.001), more meals per day (*Z *= 344, *p *< 0.001) and reduced frequency of fried food consumption (*Z *= 2.353, *p *= 0.02). During pregnancy no differences were detected with regard to consumption of particular foods among BMI groups.

**Table 3:** Differences in frequency of consumption of individual foods between BMI groups

	Pre-conceptionally								Pregnancy							
	Normal weight		Overweight an obese		*U*	*p*	Total		Normal weight		Overweight and obese		*U*	*p*	Total	
	Median	*r*_*s*_ with BMI	Median	*r*_*s*_ with BMI			Median	*r*_*s*_ with BMI	Median	*r*_*s*_ with GWG	Median	*r*_*s*_ with GWG			Median	*r*_*s*_ with GWG
Vegetables	6.0	0.164	6.0	0.069	265.0	0.365	6.0	− 0.008	6.5	− 0.019	7.0	0.050	159.5	0.770	7.0	− 0.056
Fruits	7.0	− 0.034	5.0	0.204	116.5	*0.000*	6.5	*−* 0.405*	7.0	0.186	6.0	− 0.261	98.0	0.407	7.0	0.178
Beef	2.5	− 0.206	3.0	− 0.164	407.0	*0.029*	3.0	0.111	3.0	0.034	3.0	0.291	182.0	0.331	3.0	0.025
Pork	2.0	0.381*	2.0	− 0.347	325.5	0.392	2.0	0.242	2.0	0.097	3.0	− 0.112	204.0	0.095	2.0	− 0.027
Chicken meat	3.0	− 0.153	3.0	0.188	281.5	0.638	3.0	− 0.109	3.0	0.025	3.5	− 0.173	162.5	0.701	3.0	− 0.023
Processed meat products	3.0	− 0.145	2.5	− 0.048	311.5	0.992	3.0	− 0.074	2.0	0.062	2.0	0.130	180.5	0.346	2.0	− 0.028
Eggs	3.0	− 0.191	2.5	− 0.020	189.5	*0.017*	3.0	*−* 0.341*	3.0	0.127	3.0	0.290	109.5	0.209	3.0	0.248
Milk	4.0	− 0.026	6.0	0.368	407.0	*0.046*	4.5	0.217	6.0	− 0.299	4.0	0.494	121.0	0.379	5.5	− 0.078
Cheese, cottage cheese	4.0	− 0.127	3.0	− 0.292	148.0	*0.003*	4.0	*−* 0.277*	5.0	− 0.255	4.0	− 0.222	87.5	0.414	4.0	− 0.161
Salty snacks	2.0	*−* 0.402*	2.5	*−* 0.530*	332.5	0.691	2.0	− 0.208	1.5	0.369*	2.0	0.260	156.0	0.866	*2.0*	0.325*
Sweets	3.0	− 0.100	2.5	0.012	248.0	0.225	3.0	− 0.175	2.0	0.176	2.0	0.092	151.0	1.00	2.0	0.135
Bread	6.0	− 0.190	6.0	0.508	342.5	0.315	6.0	0.044	6.0	0.096	5.5	0.000	96.0	0.479	6.0	0.176
Pasta	4.0	*−* 0.364*	3.5	0.143	300.5	0.823	4.0	− 0.192	4.0	− 0.107	3.0	− 0.518	102.0	0.140	4.0	0.019
Potato	4.0	− 0.197	4.0	− 0.051	326.5	0.770	4.0	− 0.074	4.0	0.085	3.0	− 0.632	94.0	0.105	4.0	0.162
Juice	2.0	− 0.190	1.5	− 0.024	298.0	0.788	2.0	− 0.123	1.0	0.074	1.0	0.060	160.0	0.770	1.0	− 0.003
Smoothie	3.0	− 0.045	2.0	0.171	258.5	0.308	2.0	− 0.120	2.0	0.194	1.0	0.413	130.0	0.505	2.0	0.241
Fish	3.0	0.047	2.0	− 0.190	262.5	0.299	2.0	− 0.107	3.0	− 0.065	3.0	0.082	167.0	0.225	3.0	− 0.068
Sour milk/kefir	2.0	− 0.179	2.0	0.027	244.5	0.194	2.0	− 0.227	3.0	− 0.367	2.0	0.009	138.5	0.714	2.5	− 0.227
Low calorie beverages	1.0	− 0.038	1.5	− 0.402	397.0	*0.049*	1.0	0.167	1.0	0.034	1.0		120.0	0.363	1.0	0.118

Italics text indicates statistically significant correlation or difference between groups

GWG: gestational weight gain; *r*_*s*_ with BMI: Spearman correlation with BMI; *r*_*s*_ with GWG: Spearman correlation with weight gain

*p < 0.05 level

### Diet differences among personality types

Personality types differed in lifestyle related variables only pre-conceptionally. They significantly contributed to differences on the score of regarding healthy diet principles pre-conception (χ^2^(2) = 9.290; *p *= 0.01). Analyzers, with mean rank score 9.36, followed the principles of a healthy diet to a lesser extent in comparison with facilitators, with mean rank score 22.00, *p *= 0.009 and promoters, with mean rank score 27.28, *p *= 0.04. During pregnancy there were no differences among personality types.

### Diet-related factors, associated with LGA infants

45.5% of infant’s birth weight was above 90th percentile, 14.5% weighted more than 4000 g. LGA was equally distributed across BMI groups (*p *= 0.83) (see Additional file 1: Table S2). Gestational weight gain ranged from 0.5 to 22.9 kg (10.3 ± 5.0) and was positively correlated with the infant’s birth weight (*r *= 0.330, *p *= 0.02), although no statistical significance was found after correction for gestational age.

A healthy eating score improvement during pregnancy was associated with LGA (*r *= 0.371, *p *= 0.03) while fish consumption during pregnancy was negatively associated with LGA (*r *= − 0.444, *p *= 0.008). Serum triglycerides were not significantly associated with LGA (*r *= 0.067, *p *= 0.66).

Some maternal and paternal anthropometric factors were associated with LGA. Maternal height was positively associated LGA in the normal weight group (*r *= 0.364, *p *= 0.02), whereas paternal height (*r *= 0.316, *p *= 0.02) and BMI (*r *= 0.577, *p *= 0.01) were significantly correlated with LGA in the whole sample and in the overweight/obese group, respectively.

Statistically significant variables associated with LGA (*p *< 0.05) from a univariate logistic regression model are presented in Table 4. In the multivariate logistic regression model, difference in the healthy diet score was a statistically significant predictor of the LGA in the first step (OR = 1.39, 95% CI 1.02–1.90). However, after adjustment for paternal height, employment status and glycated hemoglobin, improvement in diet was no longer a significant predictor for LGA (adjusted OR = 1.70, 95% CI 0.80–3.60).

**Table 4:** Univariate logistic regression for variables associated with LGA

	OR [95% CI]	*p* value
Paternal height	1.12 [1.01, 1.23]	0.03
Employment (unemployed)	4.80 [1.12, 20.61]	0.04
Healthy eating score	1.19 [1.02, 1.39]	0.03
HbA1c_3rd trimester_ [0.1%]	0.50 [0.26, 0.97]	0.04

## Discussion

Both, maternal obesity and GDM are risk factors, associated with obesity of the offspring as well as type 2 diabetes of the offspring and mother later in life. Therefore, optimizing treatment of pregnant women with GDM to avoid or reduce long and short term negative outcomes is of the utmost importance. The majority of participants in our study showed improvement in dietary habits during pregnancy. We showed that pre-conception diet disparities among normal weight and overweight/obese groups and among different personality types disappeared during pregnancy. Women improved their dietary patterns, independently of obesity status or personality type, thereby confirming the success of education and non-pharmacological treatment of GDM. Excessive gestational weight gain did not significantly differ between the BMI groups; neither did the incidence of LGA infants.

Many potentially modifiable variables were included in our study, which could help us adjust education to vulnerable groups. Another strength was that findings are easily transferable to our everyday clinical work with patients, because of the standard care treatment participants received. The research was also subjected to some limitations. The main disadvantage is small number of participants included and inequality in the number of participants in each group. The sample may be too small to detect statistically significant differences between groups. Voluntary participation may also bias findings. Those that decided to participate could have had a stronger interest in the main topic of our research and could have been more willing as well as more motivated to change their lifestyles. Further, major issues regarding the validity of dietary recalls are related to how accurately individuals can recall what they ate [21]. With time, these recalls are even less trustful and rely more on semantic memory, which may be more subject to a social approval bias than specific memory of specific recalls [22]. Also, social desirability may affect validity [23].

Unexpectedly, disparities among BMI groups pre-conception regarding the healthy diet score were not found in this study. Both groups followed the healthy diet guidelines. In addition, a positive association between BMI and fried food consumption was expected [24]. However, a negative correlation was found, especially among overweight/obese women. This may have been due to assessment through self-report. Overweight and obese women may be hesitant to report socially less desirable behavior, which might be often attributed to them as a cause of their obesity. Dietary studies showed that individuals who exhibited social desirability characteristics are more prone to underreport energy and fat intake [23, 25, 26]. In addition, normal weight women may pay more attention to their diet before pregnancy, which could contribute to normal BMI and therefore greater accuracy of their report. Alternatively, normal weight women might care less about what they eat and pay more attention to quantity rather than quality of the food consumed. Of note, we do not have data on the amount of food consumed, which, together with psychical activity, definitely contribute to BMI.

In our study infrequent consumption of fruits, eggs and cheese at baseline contributed to higher BMI pre-conception. Compared to the normal weight group, overweight/obese women more often consumed milk and beef before pregnancy. Our results on fruits and beef intake support the findings from other research groups [27–29]. On the other hand, the correlation between egg consumption and BMI was not universally found [30]. However, studies are not entirely comparable due to differences in the investigated populations. Moreover, existing data on the association between dairy products and BMI do not provide clear conclusions [31, 32]. It would probably be reasonable to measure the contribution of different dairy products to BMI individually, especially in relation to the development of diabetes later in life [33].

With the personality test [20] we aimed at investigating possible psychological contributions to differences in nutritional habits and possibly indirectly to obesity and gestational weight gain. However, in our study personality types differed only in relation to the principles of healthy diet pre-conception. Women who were classified as analyzers achieved the lowest score and declared as a group who ate the least healthy. The healthiest diet pre-conception was significant for promoters. Individual personality traits and eating styles may play a role in the extent to which individuals adhere to dietary guidelines and could play a role in maintaining a healthy lifestyle after pregnancy. Healthy choices of foods recommended by dietary guidelines are promoted by conscientiousness [34]. Some features such as discipline, self-control or work orientation, characteristics of consciousness, are less typical for analyzers than facilitators or promoters. In our study, differences in diet regarding personality types during pregnancy were not detected. However, personality types were not equally represented, therefore, further validations are needed.

Gestational weight gain is a well-recognized risk factor for LGA [35], but in our study the association was not confirmed. This may be due for not measuring the quantity of individual foods. As well as we did not assess all possible (important) factors contributing to gestational weight gain. Nevertheless, the percentage of women that exceeded the recommended weight gain was smaller than in most other studies, possibly due to a good follow-up at gynecologists and diabetes care clinics [36, 37].

Despite the fact that only a minority of women exceeded the recommended gestational weight gain and that all exhibited good glycemic control, the prevalence of LGA newborns in our study was relatively high. Interventions could help to limit weight gain. However, other studies also showed that their power to ameliorate effects on birth outcome is limited [38–40]. Obesity has been reported to increase the risk of LGA delivery [41], however, in our study the prevalence of LGA newborns was comparable among the two BMI groups. Interestingly, improvement in diet from pre-conception was a significant predictor for LGA. The reason may be that in women in whom accelerated fetal growth was observed during pregnancy, more intensive education and more contacts with the nurse educators was in place to improve their diet. On the other hand, it is also possible that these women just provided more socially desirable answers. This however, is less likely as these women also exhibited lower glycated hemoglobin values. Nevertheless, it is plausible that unmeasured genetic and epigenetic factors play a decisive role in the intrauterine growth regulation [42, 43].

Special attention must be paid to the high risk of occurrence of LGA among unemployed women. The link between social determinants of health and pregnancy outcomes deserves special attention in future research. Maternal education is positively associated with children’s eating habits, and parental healthy eating habits may serve as mediators [44]. Therefore, awareness of the importance of a healthy lifestyle must be raised among more vulnerable groups with the aim of gaining healthier habits and introducing them into the family environment. With the aim of closing the gap, group peer sessions, where they could make new contacts and exchange experiences, could be introduced [45]. Furthermore, the assessment of different programs for reduction of obesity postpartum is still unanswered. Monitoring and guiding obese women not just during pregnancy, but also before and after pregnancy, would probably provide the best pathway to reduction of obesity in society long-term.

## Conclusion

To sum up, in patients with GDM, diet improvement and tight glycemic control cannot completely prevent LGA infants. Instead, efforts to interrupt obesity cycle should focus more on social support and mental health, particularly among obese women, in whom clinical psychological assessment and support would be almost necessary.

## Supplementary information

**Additional file 1: Table S1.** Questionnaire on eating behavior. **Table S2.** Anthropometric and pregnancy outcome data.

## Data Availability

The datasets used and analysed during the current study are available from the corresponding author on reasonable request.

## Abbreviations

GDM: gestational diabetes mellitus
LGA: large for gestational age
OGTT: oral glucose tolerance test
BMI: body mass index
GWG: gestational weight gain
*SD*: standard deviation
