# Supplementary material for: Association between pre-pregnancy body weight and dietary pattern with large-for-gestational-age infants in gestational diabetes
Source: Diabetol Metab Syndr. 2019 Aug 22;11:68. doi: 10.1186/s13098-019-0463-5 (PMC6706911; doi:10.1186/s13098-019-0463-5)
Supplement: Supplementary file 1 — Additional file 1: Table S1. Questionnaire on eating behavior. Table S2. Anthropometric and pregnancy outcome data. [file 13098_2019_463_MOESM1_ESM.docx]

Table S1

*Questionnaire on eating behavior*

| Healthy diet principles are listed below. On the scale from 1 to 5 evaluate how much each healthy diet principle suits your lifestyle, where 1 means *totally disagree* and 5 *totally agree*. | | | | | |
| --- | --- | --- | --- | --- | --- |
| I always enjoyed my meal and ate frequently | 1 | 2 | 3 | 4 | 5 |
| I ate various food groups and my meal consisted of more plant based food sources than animal sources. | 1 | 2 | 3 | 4 | 5 |
| I was choosing whole-grain products. | 1 | 2 | 3 | 4 | 5 |
| I ate various vegetables and fruits several times per day. | 1 | 2 | 3 | 4 | 5 |
| I was choosing local fresh fruits and vegetables. | 1 | 2 | 3 | 4 | 5 |
| I was monitoring the level of fat intake and exchanged most saturated fat (animal sources) with unsaturated vegetable oils. | 1 | 2 | 3 | 4 | 5 |
| I exchanged greasy meat and greasy meat products with leguminous, fish, poultry and lean meat. | 1 | 2 | 3 | 4 | 5 |
| I consumed daily recommended quantities of less greasy milk and less greasy dairy products. | 1 | 2 | 3 | 4 | 5 |
| I ate less salty foods. | 1 | 2 | 3 | 4 | 5 |
| I limited the amount of sugar and sugary products consumed. | 1 | 2 | 3 | 4 | 5 |
| I drank enough liquids. | 1 | 2 | 3 | 4 | 5 |
| I limited alcohol consumption. | 1 | 2 | 3 | 4 | 5 |
| I prepared food in a healthy and hygienic way. | 1 | 2 | 3 | 4 | 5 |

Table S2

*Anthropometric and pregnancy outcome data*

|  | Normal weight  M±SD  n (%) | Overweight  M±SD  n (%) | Obese  M±SD  n (%) | Overweight and obese  M ± SD  n (%) | Total  M ± SD  n (%) |
| --- | --- | --- | --- | --- | --- |
| Pre gravid BMI (kg/m^2^) | 22.12±1.40 | 27.70±1.77 | 35.70±3.21 | 31.20±4.75 | 24.67±4.94 |
| Maternal gestational weight gain (kg) | 11.59±4.92 | 7.10±3.89 | 6.28±2.86 | 6.72±3.34 | 10.30±5.02 |
| Gestational age (weeks) | 38.97±1.31 | 38.75±1.39 | 39.00±1.29 | 38.87±1.30 | 38.94±1.30 |
| Infant’s birth weight (g) | 3556±407 | 3375±843 | 3534±447 | 3449±669 | 3524±497 |
| Infant’s birth weight (g) (born ≥ 37 week) | 3556±407 | 3559±712 | 3534±447 | 3546±574 | 3554±456 |
| LGA | 18 (46.2) | 4 (44.4) | 3 (42.9) | 7 (43.8) | 25 (45.5) |
| SGA | 1 (2.6) | 2 (22.2) | 0 (0) | 2 (12.5) | 3 (5.5) |
| Weight gain according to IOM guidelines |  |  |  |  |  |
| < IOM guidelines | 15 (41.7) | 4 (57.1) | 1 (16.7) | 5 (38.5) | 20 (40.8) |
| = IOM guidelines | 15 (41.7) | 1 (14.3) | 4 (66.7) | 5 (38.5) | 20 (40.8) |
| > IOM guidelines | 6 (16.6) | 2 (28.6) | 1 (16.7) | 3 (23.1) | 9 (18.4) |
| Hba1c _2ndtrimester_ (%) | 4.96±0.06 | 4.90±0.13 | 5.10±0.10 | 4.96±.10 | 4.96±0.29 |
| HbA1c _3rd trimester_ (%) | 5.11±0.39 | 5.37±0.12 | 5.18±0.13 | 5.23±0.15 | 5.15±0.34 |
| Triglycerides (mmol/l) | 1.77±0.62 | 2.11±0.60 | 1.74±0.40 | 1.97±0.55 | 1.83±0.60 |

†LGA = infants, overweight for gestational age (>90.^th^percentile).

‡SGA = infants, too small for gestational age (<10^th^ percentile).
